# The association of duration of boarding in the emergency room and the outcome of patients admitted to the intensive care unit

**DOI:** 10.1186/s12873-017-0143-4

**Published:** 2017-11-09

**Authors:** Saad Al-Qahtani, Abdullah Alsultan, Samir Haddad, Abdulmohsen Alsaawi, Moeed Alshehri, Sami Alsolamy, Afef Felebaman, Hani M. Tamim, Nawfal Aljerian, Abdulaziz Al-Dawood, Yaseen Arabi

**Affiliations:** 1 Intensive Care Department, King Abdulaziz Medical City, P.O. Box 22490, Riyadh, 11426 Saudi Arabia; 2 King Saud bin Abdulaziz University for Health Sciences, Riyadh, Saudi Arabia; 3 King Abdullah International Medical Research Center, Riyadh, Saudi Arabia; 4 Emergency Medicine Department, King Abdulaziz Medical City, Riyadh, Saudi Arabia; 5 Emergency Medicine and Intensive Care Department, College of Medicine, King Abdulaziz Medical City, Riyadh, Kingdom of Saudi Arabia; 6 Obstetrics and Gynecology Department, King Abdulaziz Medical City, Riyadh, Saudi Arabia; 7 Department of Internal Medicine, American University of Beirut- Medical Center, Beirut, Lebanon

**Keywords:** Emergency service, hospital, ED boarding, ICU, Critical illness, Retrospective studies, Length of stay, Hospital mortality, Critical care, Saudi Arabia

## Abstract

**Background:**

The demand for critical care beds is increasing out of proportion to bed availability. As a result, some critically ill patients are kept in the Emergency Department (ED boarding) awaiting bed availability. The aim of our study is to examine the impact of boarding in the ED on the outcome of patients admitted to the Intensive Care Unit(ICU).

**Methods:**

This was a retrospective analysis of ICU data collected prospectively at King Abdulaziz Medical City, Riyadh from ED between January 2010 and December 2012 and all patients admitted during this time were evaluated for their duration of boarding. Patients were stratified into three groups according to the duration of boarding from ED. Those admitted less than 6 h were classified as Group I, between 6 and 24 h, Group II and more than 24 h as Group III. We carried out multivariate analysis to examine the independent association of boarding time with the outcome adjusting for variables like age, sex, APACHE, Mechanical ventilation, Creatinine, Platelets, INR.

**Results:**

During the study period, 940 patients were admitted from the ED to ICU, amongst whom 227 (25%) were admitted to ICU within 6 h, 358 (39%) within 6–24 h and 355 (38%) after 24 h. Patients admitted to ICU within 6 h were younger [48.7 ± 22.2(group I) years, 50.6 ± 22.6 (group II), 58.2 ± 20.9 (group III) (*P* = 0.04)]with less mechanical ventilation duration[5.9 ± 8.9 days (Group I), 6.5 ± 8.1 (Group II) and 10.6 ± 10.5 (Group III), *P* = 0.04]. There was a significant increase in hospital mortality [51(22.5), 104(29.1), 132(37.2), *P* = 0.0006) and the ICU length of stay(LOS) [9.55 days (Group I), 9.8 (Group II) and 10.6 (Group III), (*P* = 0.002)] with increase in boarding duration. In addition, the delay in admission was an independent risk factor for ICU mortality(OR for group III vs group I is 1.90, *P* = 0.04) and hospital mortality(OR for group III vs Group I is 2.09, *P* = 0.007).

**Conclusion:**

Boarding in the ED is associated with higher mortality. This data highlights the importance of this phenomenon and suggests the need for urgent measures to reduce boarding and to improve patient flow.

## Background

Shortage in Intensive care unit(ICU) beds is an increasing challenge worldwide [1]. The demand for critical care beds is increasing out of proportion to bed availability [2], as a reflection of aging of the population and increase in the complexity of medical disease. Additionally in Saudi Arabia, the high prevalence of motor vehicle accidents increases the pressure on the existing ICU bed capacity [3, 4]. As a result, some critically ill patients are kept in the Emergency Department (ED boarding) awaiting bed availability. ED boarding is common and increasing worldwide, resulting in a prolonged length of stay [5]. The waiting time for ICU admission varies between hospitals and nations [6] from two hours to few days [2, 5–8]. ED boarding can have substantial consequences leading to delay in time-sensitive interventions. For example, early goal- directed therapy if initiated as soon as possible in severe sepsis and septic shock shows a significant improvement with respect to outcomes(hospital mortality) [9]. Administration of thrombolytic agents in acute ischemic stroke is recommended within 3 h of the symptom onset [10]. Additionally, boarding in the ED has become a significant barrier to specialized critical care [11] and may jeopardize patients safety when there is not enough staffing in the ED increasing medical errors [12]. ED boarding has been used as a surrogate for adverse outcomes [5]. There are conflicting data in the association between delayed admission and outcome. There have been only a few studies examining the association of boarding and outcome [6, 13]. The aim of our study is to examine the association of boarding duration in the ED and the outcome of patients admitted to the Intensive Care Unit.

## Methods

The study was a retrospective cohort study conducted in a 900-bed tertiary care teaching hospital in Riyadh, Saudi Arabia. The study was approved by the Institutional Review Board of the Ministry of National Guard Health Affairs. The Emergency department receives around 200,000 visits per year. Critically ill patients are referred to the ICU team. Due to ICU bed shortage, these patents are managed by the critical care team which is led by a board certified intensivist in the ED. Upon availability of ICU beds, patients are then transferred to the ICU. These ICUs admit medical, surgical, and trauma patients, and operate as a closed unit with 24-h, 7-day onsite coverage by critical care board certified intensivists. The nurse-to-patient ratio in the unit is approximately 1:1.2. Many patients however are managed and sign off in the ED.

### Study population

This study included all the consecutive patients who were admitted to ICUs from the ED between January 2010 and December 2012. Our ICU admits all the patients >16 years age. We excluded patients who were admitted from ED after emergency surgery because they were transferred immediately to the operation theatre(OT). In addition, elective admissions were also excluded because they occurred mainly during weekdays and typically have a lower severity of illness. Patients were stratified into three groups according to the duration of boarding in the ED. Duration of boarding was defined as the length of stay of the patients from ED admission to ICU admission. Those admitted less than 6 h were classified as Group I, between 6 and 24 h as Group II and more than 24 h as Group III.

### Data collection

Data of critically ill patients in the ED admitted to ICU were prospectively collected and entered in an electronic ICU database which consists of all consecutive admissions with data being collected prospectively by a full-time data collector. We extracted the following data: baseline demographics including age, gender, Glasgow coma score (GCS), serum creatinine, and prespecified admission diagnoses. Acute Physiology and Chronic Health Evaluation II (APACHE II) scores [14] and chronic co-morbidities (chronic liver disease, chronic cardiovascular disease, chronic respiratory disease, chronic renal disease and chronic immunosuppression) as defined by APACHE II system were also included. We grouped the main reasons for ICU admissions based on APACHE II system into the following categories: respiratory, cardiovascular, neurological, other medical, non-operative trauma and post-operative.

### Endpoints

The primary endpoints of our study were the hospital mortality and ICU mortality. ICU length of stay (LOS), hospital LOS, total LOS (ICU and ED) and the mechanical ventilation duration (MV) were secondary outcomes.

The Statistical Analysis Software (SAS), version 9.1, was used for the data management and analyses. Continuous data were expressed as means and standard deviation (SD) and compared using the Analyses of Variance (ANOVA), whereas the categorical data were expressed as number- percentage and compared using the chi-square test. Stepwise multivariate logistic regression analyses (for categorical outcomes) and stepwise multivariate linear regression analyses (for continuous outcomes) were carried out to examine the independent association of boarding time with the outcome adjusting for variables like age, sex, APACHE, Mechanical ventilation, Creatinine, Platelets, INR which were either found to be statistically significant in univariate analysis or were clinically significant. Statistical significance was defined as an alpha less than 0.05.

## Results

### Demographic and baseline characteristics

Over three years, 940 patients were admitted from the ED to the ICU, amongst whom 227 (25%) were admitted to ICU within 6 h (group I), 358 (39%) within 6–24 h (group II) and 355 (38%) after 24 h (group III). At baseline, the three groups had stepwise differences in several variables. Patients admitted to ICU within 6 h were significantly younger [48.7 ± 22.2(group I) years, 50.6 ± 22.6 (group II), 58.2 ± 20.9 (group III) (*P* = 0.04)] and required less frequently mechanical ventilation [141(62.1%), 269(75.1%), 257 (72.4%) (*P* = 0.003]. The main indication for admission was cardiac followed by respiratory, neurological and other medical conditions. There was no significant difference in chronic comorbidities between the three groups except chronic respiratory disease[19 (8.4%), 38 (10.6%), 71 (20.0%) *P* = <0.0001] which included chronic obstructive, restrictive or vascular disease, chronic hypoxia, hypercapnia, severe polycythemia and severe pulmonary hypertension (Table 1).

**Table 1:** Baseline Characteristics of patients stratified into three groups based on boarding time

Variables	Group I (<6 h) *N* = 227	Group II (6–24 h) *N* = 358	Group III (>24 h) *N* = 355	*P*- value
Age - yr. (mean ± SD)	48.7 ± 22.2	50.6 ± 22.6	58.2 ± 20.9	0.04
Gender - no. (%)				
Female	37 (20.4)	48 (16.9)	76 (26.4)	0.02
Male	144 (79.5)	235 (83.0)	212 (73.6)	
APACHE score - (mean ± SD)	19.4 ± 9.7	20.1 ± 9.8	21.8 ± 8.2	0.38
Mechanical ventilation, no. (%)	141 (62.1)	269 (75.1)	257 (72.4)	0.003
GCS - (mean ± SD)	11 ± 4.2	11 ± 4.2	10.7 ± 4.2	0.87
PaO_2_/FiO_2_ - (mean ± SD)	260 ± 138.9	223.8 ± 122.6	207.3 ± 90.9	0.38
Admission category, no. (%)				
Non-operative	220 (96.9)	353 (98.6)	347 (97.8)	0.38
Post-operative	7 (3.1)	5 (1.4)	8 (2.3)	
Admission Diagnosis Category, no. (%)				
Respiratory	40 (17.6)	94 (26.3)	122 (34.4)	<0.0001
Cardiovascular	63 (27.8)	96 (26.9)	129 (36.3)	
Neurological	49 (21.6)	53 (14.9)	32 (9.0)	
Other medical	15 (6.6)	30 (8.4)	23 (6.5)	
Non-operative trauma	53 (23.4)	79 (22.1)	41 (11.6)	
Post-operative	7 (3.1)	5 (1.4)	8 (2.3)	
Chronic comorbities, no. (%)				
Chronic respiratory	19 (8.4)	38 (10.6)	71 (20.0)	<0.0001
Chronic cardiac	30 (13.2)	61 (17.0)	71 (20.0)	0.11
Chronic renal	20 (8.8)	34 (9.5)	41 (11.6)	0.50
Chronic liver	11 (4.9)	19 (5.3)	28 (7.9)	0.23
Chronic Immunosuppression	14 (6.2)	21(5.9)	31 (8.7)	0.28
Lab findings				
Creatinine - (mean ± SD) (μmol/L)	153.3 ± 197.1	161.6 ± 185.5	174.1 ± 152.5	0.11
Platelets - (mean ± SD) (10^9^/L)	211 ± 108.5	202.8 ± 112.5	20.7 ± 137.3	0.132
INR	1.5 ± 0.8	1.94 ± 1.50	1.5 ± 0.9	0.004

*APACHE II* Acute Physiology and Chronic Health Evaluation II, *GCS* Glasgow coma scale, *PaO2/FIO2 ratio* the ratio of partial pressure of oxygen to the fraction of inspired oxygen, *INR* international normalized ratio, *SD* Standard deviation

### Outcomes

Hospital mortality was lower in group I and it increased with the increase in boarding duration {51(22.5), 104(29.1), 132(37.2), *P* = 0.0006). There was also a significant increase in the ICU LOS with increase in boarding duration [9.6 days (Group I), 9.8 (Group II) and 10.6 (Group III), (*P* = 0.002)]. The duration of mechanical ventilation was longer with increase in boarding [5.9 ± 8.9 days (Group I), 6.5 ± 8.1 (Group II) and 10.6 ± 10.5 (Group III), *P* = 0.04], (Table 2).

**Table 2:** Outcomes of patients stratified into three groups based on boarding time

Variables	Group I (<6 h) *N* = 227	Group II (6–24 h) *N* = 358	Group III (>24 h) *N* = 355	*P*- value
ICU mortality **-** no. (%)	41 (18.1)	78 (21.8)	89 (25.2)	0.13
Hospital mortality **-** no. (%)	51(22.5)	104 (29.1)	132 (37.2)	0.0006
ICU LOS – (mean ± SD)	9.5 ± 10.2	9.8 ± 12.3	10.6 ± 10.5	0.0023
Hospital LOS – (mean ± SD)	26.7 ± 33	25.1 ± 27.5	21.5 ± 223.4	0.29
Ventilation duration **-**(mean ± SD)	5.9 ± 8.9	6.5 ± 8.1	10.6 ± 10.5	0.04
Total LOS(ICU + ER)**-** (mean ± SD)	9.6 ± 10.2	10.4 ± 12.3	14.3 ± 12.0	<0.0001

*ICU* Intensive care unit, *LOS* Length of stay, *ER* Emergency room

Multivariate analysis demonstrated that the duration of boarding was associated with the increased risk of ICU mortality in group III compared to group I [odds ratio 1.9; 95% confidence interval (CI) 1.02 to 3.54, *P* = 0.04] and also the duration of boarding in group III compared to group I was associated independently with increased hospital mortality [odd ratio 2.09; 95% CI 1.22 to 3.6, *P* = 0.007]. Similarly there was a significant increase in the total LOS (ICU + ER) with increase in the boarding time in group III compared to group I [Estimate 4.18; 95% CI 2.04 to 6.2, *P* < 0.001], (Table 3).

**Table 3:** Multivariate analysis to examine the independent association of boarding time with the outcome

Outcome	Group I	Group II		Group III	
		OR (95% CI)	*P* value	OR (95% CI)	*P* value
ICU mortality	Ref	1.62 (0.86, 3.10)	0.13	1.90 (1.02, 3.54)	0.04
Hospital mortality	Ref	1.54 (0.90, 2.70)	0.12	2.09 (1.22, 3.60)	0.007
		Estimate		Estimate	
ICU LOS	Ref	1.15 (−0.76, 3.11)	0.24	0.84 (−1.09, 2.77)	0.39
Hospital LOS	Ref	−2.50 (−40.43, 35.42)	0.87	−11.76 (−48.67, 25.15)	0.46
Total LOS (ICU + ER)	Ref	1.68 (−0.34, 3.83)	0.12	4.18 (2.04, 6.21)	<0.0001

Adjusted variables: age, sex, APACHE, Mechanical ventilation, Creatinine, Platelets, INR, *OR* Odds ratio

## Discussion

Our data highlights the importance of ED boarding and suggests the need for urgent measures to reduce boarding to improve the patient flow.

The shortage of ICU beds is a worldwide dilemma even in the United States. It has been reported that there is more than six-hour delay in admission to ICU. Few studies focused on ED boarding to ICU. The phenomenon of waiting for ICU bed availability has been suggested to be associated with increased mortality. This may be a reflection of the fact that the other health care providers are not trained in the critical care and that the ED is neither designed nor staffed to provide extended care to critically ill patients [12].

In concordance to our finding a Brazilian study examined 401 admissions and found delay in ICU admission by median of 17.8 h. Patients in this study, were managed by ward staff while waiting for a ICU bed and the delayed admission to ICU was associated with 1.5% increased risk of ICU death for each 1 h delay [6]. A study from United States examined more than 50,000 patients from database of approximately 120 ICUs and found that delayed admission for more than six hours was associated with increased hospital LOS and higher ICU and hospital mortality. However the incidence of delayed admission was only 2% [5]. In a study that analyzed four prospective cohort studies from North America and Europe of adult patients with community acquired pneumonia, the authors compared patients admitted directly from ED (no-delay) to ICU with patients admitted first to ward (delay). The study found that after adjusting for propensity score, delayed ICU transfer was associated with increased odds ratio for 28-day mortality and longer median hospital length of stay [15]. Another Australian study found that patients admitted to ICU within 24 h of ward admission from ED had a significant increase in 30 day mortality compared with patients admitted to ICU directly from ED [16]. A study from community hospital showed increased mortality associated with delay of more than four hours in transfer to ICU [17]. Simchen and his colleagues showed improved early survival for ICU patients compared with patients treated in regular departments [1].

In contradiction to our findings, a study in United Kingdom by O’Callaghan et al. showed that delayed admission to ICU by more than three hours did not affect the ICU length of stay, ICU and hospital mortality [13].However, this study included the large percentage of patients in the delayed group admitted from OT interestingly and showed a low rate of mortality compared to the patients managed in ED [13]. However, in our study, we did not include these patients, because they form a distinct group. Another study from a single center in the United States found the patients remaining in the ED for more than 24 h did not have increased mortality [18]. In a Finnish study, they found the delay ED admission to ICU was not associated with worse outcome, and not on the health-related quality of life (HRQoL) at 6 months after ICU admission [19].

It appears that the incidence of delay in ICU admission varies considerably among studies from different countries from 69% in a south America [6] to 33%–38% in Europe [20, 21] and 2% in North American studies [5].This may be explained in part by the large variations in intensive care unit bed provision among countries [13]. Differences between our finding and those of previous findings may be explained by selection bias of triaging the critically ill patients. It has been recommend by the American Thoracic Society(ATS) members that when demand for ICU beds exceeds supply, medically appropriate patients should be admitted on a first-come, first-serve basis [22]. The priority to ICU admission depends on need of special interventions such as hemodialysis or high ventilation setting in addition to young patient and revivable diseases. In addition, within our institution there is an ICU team led by an ICU consultant who manages critically ill patients in the ED boarding. Even though there is no difference in the APACHE II score, this could be explained by the fact that the sample of patients of both groups for whom APACHE II were calculated did not reflect the acuity of illness in the cohort [18]. Our center is also accepting all referrals from ED whereas a study from France shows the ICU refusal rate of more than one fifth of the referral and in UK more than one sixth of ICU referrals were refused [21]. The refusal depended on both patients and organizational factors [20].

There is an evidence that in disease states like septic shock the early goal directed therapy within the first six hours can decrease the mortality [9]. This data highlights the importance of this phenomenon and suggests the need for urgent measures to reduce boarding and to improve patient flow. As ICU bed utilization continues to rise and is not able to meet the growing demand, the phenomena of boarding of critically ill patients in the ED will continue to rise unless there is expanding in the ICU beds in overall to meet the demand. In UK, it was estimated that a two-fold increase in the number of ICU beds was required to meet the demand [23].Therefore our institution requires significant increase in the number of ICU beds since the delay of admission to ICU beds is very high. Although, early transfer to ICU is beneficial for critically ill patients, we also need to focus on the timely instigation of specific interventions and organ support. These do not always mandate immediate ICU admission and can be instigated on alternate sites, such as the ED or OT, while an ICU bed is made available. Thus alleviating the ER boarding and initiating treatment immediately will require a multidisciplinary system-wide approach.

Our study has several limitations. First we analyzed data from a single center. Second, the nature of the observational study could not access some potentially significant predicators of outcome (e.g. prognosis of the critically ill patients at the presentation to the ED). Third, the result may have been influenced by the selection bias of triage patients to the ICU. In addition the sample size was not large.

## Conclusion

Our study showed that the duration of boarding due to unavailability of ICU beds is a common occurrence and occurred in 78% of ED admissions to ICU. The study demonstrated an association between the duration of ED boarding of more than twenty four hours with higher hospital mortality, duration of mechanical ventilation as well as increased total LOS.

## Abbreviations

APACHE II: Acute Physiology and Chronic Health Evaluation II
ED: Emergency department
GCS: Glasgow coma scale
ICU: Intensive care unit
LOS: Length of stay
MV: Mechanical ventilation
OT: Operation theatre
